# Continental vs. Insular: Demographic and Growth Patterns in *Acanthodactylus schreiberi*

**DOI:** 10.3390/ani15040474

**Published:** 2025-02-07

**Authors:** Büşra Kara, Mehmet Zülfü Yıldız, Deniz Yalçınkaya, Abdullah Altunışık

**Affiliations:** 1 Biology Department, The Institute of Graduate Studies, University of Recep Tayyip Erdoğan, Rize 53100, Türkiye; 2 Zoology Section, Biology Department, Faculty of Arts and Sciences, Adıyaman University, Adıyaman 02040, Türkiye; 3 Medical Laboratory Techniques Program, Department of Medical Services and Imaging, Vocational School, Toros University, Mersin 33140, Türkiye; 4 Biology Department, Faculty of Arts and Sciences, University of Recep Tayyip Erdoğan, Rize 53100, Türkiye

**Keywords:** population ecology, skeletochronology, sexual dimorphism, island, mainland, growth dynamics, survival rates

## Abstract

This study examines the demographic parameters, body size, survival rates, and growth dynamics of *Acanthodactylus schreiberi* populations in continental (Botaş, Türkiye) and insular (Dipkarpaz, Northern Cyprus) habitats to understand how ecological constraints influence life-history traits. By integrating skeletochronological and morphometric analyses, we identified significant interpopulation differences. The continental population exhibited broader age ranges, higher survival rates, and less restricted growth, likely due to greater resource availability. In contrast, the insular population showed life-history variations associated with limited resources, including smaller body sizes, lower survival rates, and distinct growth patterns. Sexual dimorphism was more pronounced in the insular population, with males exhibiting higher growth rates. These findings suggest that habitat size, resource availability, and isolation shape demographic and morphological traits, highlighting the evolutionary and ecological pressures in different environments. The results underscore the need for conservation strategies tailored to these unique populations, particularly in the face of environmental changes.

## Simple Summary

Understanding how environmental conditions shape the life-history traits of animals is essential for ecology and conservation. This study examines differences in body size, growth patterns, and survival rates between two populations of Schreiber’s fringe-fingered lizard (*Acanthodactylus schreiberi*): one living in mainland Türkiye and the other on the island of Northern Cyprus. Using bone analysis techniques, we determined the age and growth rates of individuals from both populations. Results reveal significant interpopulation differences; the continental population exhibits broader age ranges, higher survival rates, and less constrained growth due to resource availability, whereas the insular population shows life-history adjustments to resource-limited conditions, including distinct body size patterns and potentially earlier reproductive investment, which may compensate for reduced survival. Sexual dimorphism is more pronounced in insular populations, with males displaying higher growth rates. These findings underscore the impact of ecological constraints on life-history traits and contribute to conservation strategies for reptilian species in different habitats.

## 1. Introduction

The study of demographic and growth parameters in reptilian populations offers valuable insights into the ecological and evolutionary mechanisms shaping their life history traits [1]. Among these traits, age determination, growth patterns, and sexual dimorphism provide invaluable insights into survival strategies, reproductive biology, and population viability [2,3]. These factors are particularly relevant for species inhabiting fragmented or geographically isolated regions, where localized environmental pressures can drive distinct adaptations [1].

Continental and insular populations of the same species often exhibit marked differences in demographic structure, body size, survival rates, and growth dynamics, influenced by ecological factors such as resource availability, predation pressure, and habitat stability [4,5]. Islands, in particular, present unique ecological constraints that may drive distinct evolutionary adaptations, including shifts in body size and sexual dimorphism [6,7,8]. The ’island rule’ describes a pattern where small-bodied species tend to evolve larger sizes on islands, while large-bodied species exhibit dwarfism, likely driven by factors such as resource availability, predation pressure, and ecological release [9]. Sexual dimorphism is a widespread phenomenon in reptiles, often influenced by ecological factors [10,11]. However, its manifestation can vary between populations of the same species, reflecting different selective pressures acting in distinct environments [12,13]. Understanding these variations can shed light on the adaptive strategies employed by lizard populations to optimize survival and reproduction under varying environmental pressures [9,14].

Lizards of the genus *Acanthodactylus* are widely distributed in the Palearctic region, occupying diverse habitats ranging from arid and semi-arid ecosystems to Mediterranean environments [15]. Among them, *Acanthodactylus schreiberi*, commonly known as Schreiber’s fringe-fingered lizard, is an excellent model for examining the interplay between environmental conditions and population dynamics due to its insular and continental geographic population and adaptability. Despite its ecological significance, *A. schreiberi* is classified as Endangered (EN) on the IUCN Red List due to threats from coastal urbanization, habitat destruction, and pollution until June 2023 [16], but was listed in the Least Concern (LC) category at the last assessment [17]. The species is not currently subject to any significant threats. However, potential future impacts may arise from coastal tourist development and the paving over of sandy tracks [18]. Additionally, according to the latest taxonomic evaluations, it is concluded that *A. schreiberi* is endemic to Cyprus, and the Turkish population was introduced from Cyprus [19,20]. Considering the anthropogenic transport of the Turkish *A. schreiberi* population from Cyprus, it will be a good model to determine the environmental effects on two populations of the same origin, as this transport has not occurred in a long time. While limited studies exist on its age structure in Türkiye [21], recent research has begun to address this gap by providing foundational data on age and growth parameters.

Lizard species often exhibit sexual size dimorphism (SSD), where males and females differ in body size due to the influence of natural and sexual selection [22]. In many lizards, male-biased SSD is common and is associated with factors such as intrasexual competition, where larger males have an advantage in territorial disputes or mate acquisition [23,24]. Conversely, female-biased SSD is often attributed to fecundity selection, where larger females are capable of producing more or larger eggs, thus enhancing reproductive success [12]. Body size plays a critical role in determining the extent and type of SSD in lizards. For instance, in species with strong male-male competition, males may evolve to be larger than females, maximizing their chances of reproductive success [25]. The factors driving SSD in lizards are influenced by both intrinsic (e.g., growth rates, energy allocation) and extrinsic (e.g., resource availability, predation pressure) ecological variables [12]. For example, SSD patterns may vary between populations in response to localized environmental conditions, such as habitat type and resource distribution [26]. The integration of morphometric and demographic data is crucial for elucidating these patterns and their implications for species conservation.

This study builds upon these findings, offering the first comparative analysis of *A. schreiberi* populations from both continental (Türkiye) and insular (Cyprus) habitats. By examining demographic parameters, survival rates, body size, growth dynamics, and adult life expectancy, this research provides preliminary insights into these aspects of the species. The aim of this study is to investigate whether continental and insular populations of *A. schreiberi* exhibit differences in age structure, body size, weight, growth dynamics, survival rate, adult life expectancy, and sexual dimorphism, thereby providing insights into how varying ecological conditions influence the life history traits of this lizard species. Specifically, we hypothesize that the continental population, characterized by greater resource availability and more stable environmental conditions, will exhibit higher survival rates and less constrained growth compared to the insular population, where limited resources and environmental challenges prevail [27,28]. Additionally, we explore sexual dimorphism in body size and growth rates, seeking to identify patterns that may reflect differential selection pressures between sexes within each population [29]. Furthermore, the findings provide a foundation for reassessing conservation priorities for these populations, emphasizing the importance of preserving their unique ecological and evolutionary characteristics.

## 2. Materials and Methods

### 2.1. Species and Study Sites

**Figure 1:**
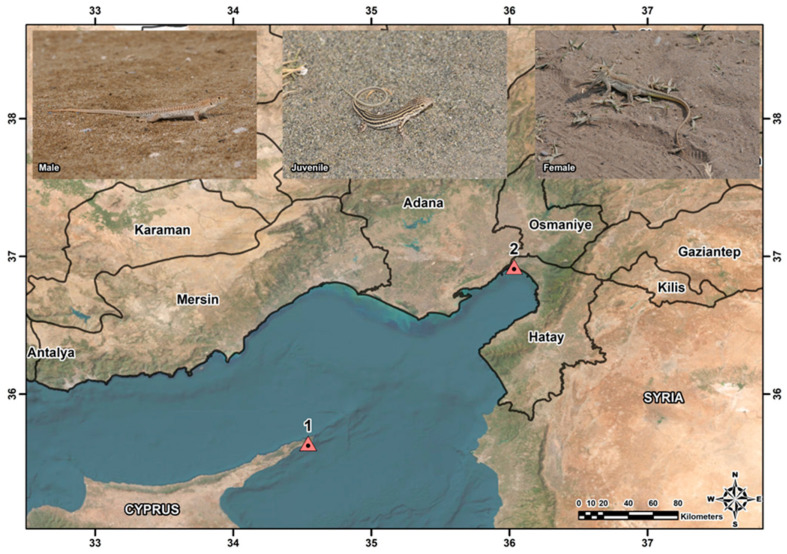
Sampling locations of the Schreiber’s fringe-fingered lizard, *Acanthodactylus schreiberi* in this study. 1: Dipkarpaz, Cyprus, 2: Botaş, Türkiye.

The samples used in this study were obtained from the Zoology Museum of Adıyaman University (ZMADYU), collected in previous studies (Supplementary File). No new living specimens were collected from the wild for this investigation. A total of 81 *Acanthodactylus schreiberi* specimens (25 females, 17 males, and 1 juvenile for continental sites; 20 females, 9 males, and 9 juveniles for insular sites) were used in this study (Figure 1).

Dicle University Health Sciences Application and Research Centre Sampling gave authorization (decision number: E-35582840-020-705112) for sampling.

The habitats of *A. schreiberi* populations in two distinct regions, Türkiye (Botaş) and Northern Cyprus (Dipkarpaz), were analyzed for their ecological characteristics. Fieldwork was conducted in both areas to gather data on sympatric species and environmental conditions. The BOTAŞ region, located near Yumurtalık, Türkiye, is characterized by abundant freshwater sources and, as a result, probably greater diversity and quantity of insects (pers. obs.), which provide an ample food supply for *A. schreiberi*. Sympatric species observed in the area include amphibians such as *Ommatotriton vittatus*, *Bufotes viridis*, and *Hyla savignyi*. Reptiles cohabiting the area include turtles like *Caretta caretta*, *Chelonia mydas*, and *Testudo graeca*, as well as lizards like *Stellagama stellio*, *Hemidactylus turcicus*, and *Ophisops elegans*. Snakes such as *Eryx jaculus*, *Macrovipera lebetina*, and *Malpolon insignithus* were also documented [30].

The Dipkarpaz area in Northern Cyprus is a semi-arid region with limited freshwater resources and, consequently, probably less abundant prey availability compared to BOTAŞ [31]. Sympatric species in this habitat include amphibians like *Bufotes viridis* and reptiles such as *Caretta caretta*, *Chelonia mydas*, *Testudo graeca*, and lizards including *Stellagama stellio*, *Hemidactylus turcicus*, and *Ophisops elegans*. Observations also noted the presence of snakes such as *Dolichophis jugularis* and *Macrovipera lebetina* [32]. Climatic information for both regions was obtained from the Meteoblue historical weather archives. For BOTAŞ, data from Yumurtalık (Türkiye) were analyzed, revealing a humid environment conducive to insect proliferation. In contrast, climate data for Dipkarpaz (Northern Cyprus) highlighted a drier environment with reduced precipitation and higher temperatures, limiting water sources and prey density [33]. Historical weather records for Yumurtalık and Rizokarpaso provided the foundational climatic comparison, accessible at Meteoblue archives for Yumurtalık and Rizokarpaso, respectively [34,35].

Males are distinguished by having larger femoral pores with filled pheromones and a red color on the ventral side of the tail during the breeding season. Females are distinguished by having smaller femoral pores without pheromones and a yellow color on the ventral side of the tail during the breeding season [30,31,36]. However, the existence or lack of hemipenis was used to determine sex [37]. The primary characteristic of color patterns that sets juveniles apart from adults is the yellowish-white stripes on a black background along the dorsal region (Figure 1). These stripes are absent in adults, being replaced by more irregular patterns. The ventral side of juveniles is dirty white. In adults, the color of the underside of the tail is related to sex and reproductive period, with reddish coloration in males and yellowish-green coloration in females [31]. A digital caliper (Mitutoyo, Japan) was used to measure the specimens’ SVL. The right hind limb’s toe was then removed and preserved in a 70% ethanol solution in accordance with Smirina’s [38] instructions.

### 2.2. Age Determination

A widely used technique for examining the age distribution of many ectotherm animals is skeletochronology. It is based on observing what are known as “growth markers” or “Lines of Arrested Growth” (LAGs), which appear in phalanges, femurs, tibias, and humerus, among other bone types. These markers are caused by reduced metabolic activity in bone tissue during inactivation or hibernation [39].

**Figure 2:**
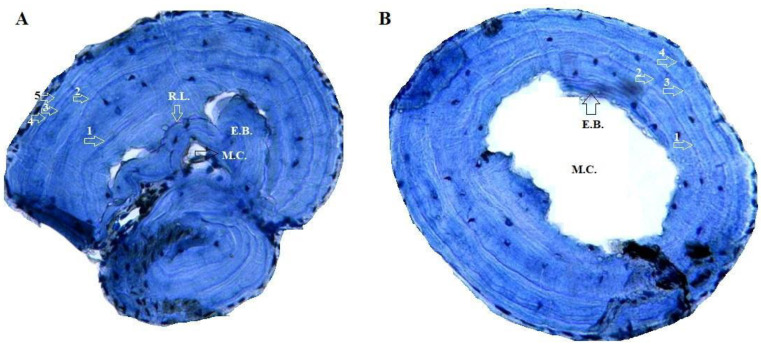
Cross-sections (18 μm thick) at the diaphysis level of the phalange bone of *Acanthodactylus schreiberi* specimens from the continental (**A**) and insular sites (**B**). Arrows indicate Lines of Arrested Growth (LAGs). E.B.: Endosteal bone; M.C.: marrow cavity; R.L.: Resorption line.

Altunışık et al.’s [40] modified procedures were used to perform the skeletochronological examination, adhering to Smirina’s [38] methodology. After being preserved in 70% ethanol, the second phalanx was immersed in distilled water for a day before being decalcified with 5% HNO_3_ for around two hours. Using a Shandon Cryostat microtome, cross-sections of 18 µm in thickness were obtained. Ehrlich’s hematoxylin dye was then added, and the samples were left for 10 min. A glycerine solution was used to carefully select sections with the smallest bone marrow cavities in order to avoid or minimize the effects of endosteal resorption, a potential cause of destruction for the innermost LAGs [41]. The chosen samples were examined and captured on camera using a light microscope fitted with a Pixera digital camera at 10x and 20x magnifications (Figure 2). Following a thorough review of all the available photographs, the authors independently calculated and verified the LAGs [42].

### 2.3. Statistics and Software

SPSS version 29.00 was used to statistically analyze the experimental data. The normality of the data was evaluated by means of the Shapiro–Wilk test, and parametric and non-parametric tests (e.g., Mann–Whitney U or *t*-test) were used to compare genders or populations. The relationship between SVL and age was investigated using Pearson’s correlation coefficient. The Sexual Dimorphism Index (SDI), as formulated by Lovich and Gibbons [43], was utilized to estimate sexual dimorphism differences.

SDI = ((size of larger sex)/(size of smaller sex)) − 1,(1)

The survival rates (Svr) were determined using the approach described by Robson and Chapman [44].

Svr = T/((R + T − 1))(2)

This calculation assumes a constant survival rate (Svr) and employs the formula T = n1 + 2n2 + 3n3 + 4n4….. R = Σ ns, and ns = number of specimens in each age group. The method takes into account the annual survival rate constraints.

The distance between two adjacent LAGs is a reliable indicator of individual growth in a given year. When a significant decrease in the distance between two successive LAGs was detected, this was taken as the age of sexual maturity [45]. The method of Seber [46] was used to calculate adult lifespan (ESP), the expected lifespan of animals that reach sexual maturity.

ESP = 0.5 + 1/(1 − Svr)(3)

Following the methodology established in earlier studies [40,47], the von Bertalanffy growth model was utilized to describe and quantify growth dynamics. The model is typically represented in its general form as:

SVLc = SVLmax (1 – e − k(c − t0))(4)

In the given formula, “SVLc” denotes size at age c, “SVLmax” represents the highest asymptotic SVL, “e” denotes Euler’s number (2.718), “k” defines the growth coefficient shaping the curve, and ’t0’ denotes the metamorphosis age.

The MS Excel program was utilized to calculate SVLmax, k, and growth rates, employing the formula “r = k (SVLmax − SVLt)”. To investigate variations in growth rates both within and between populations, we conducted *t*-tests.

## 3. Results

### 3.1. Demographic Parameters and Body Size in the Continental Population (Botaş, Türkiye)

**Figure 3:**
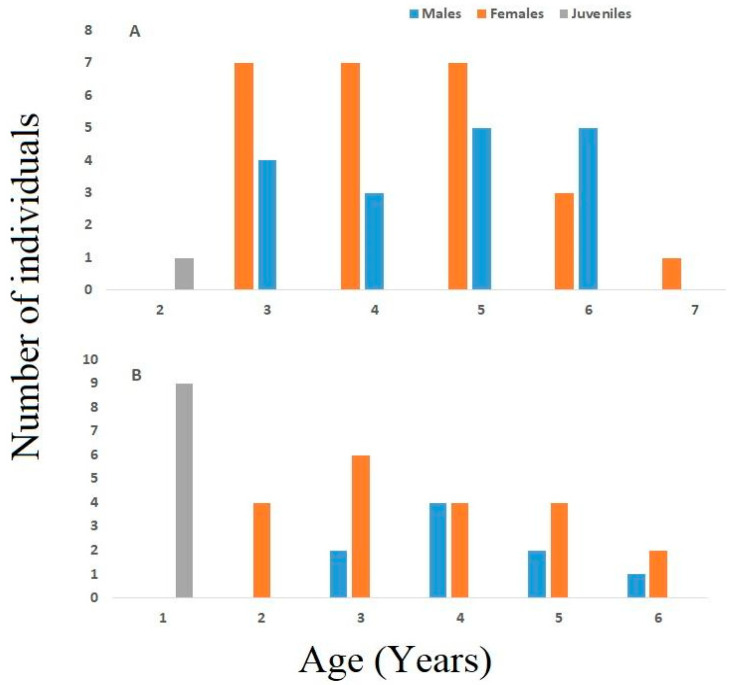
Age distribution in the continental (**A**) and insular (**B**) populations of *Acanthodactylus schreiberi*.

**Figure 4:**
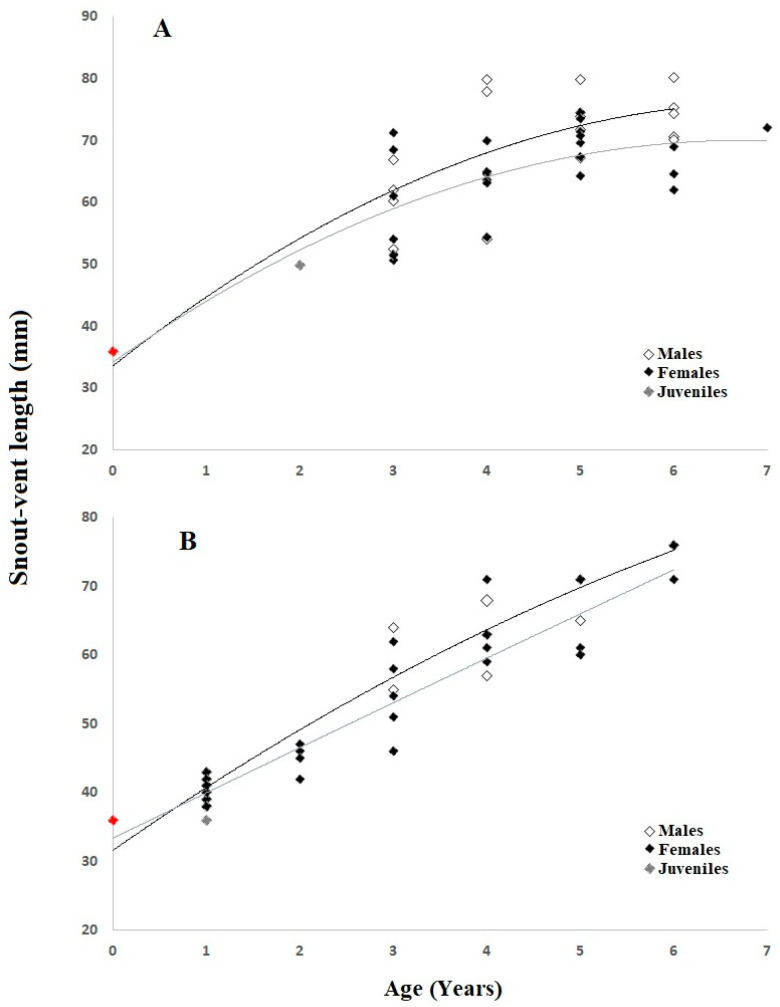
Relationship between SVL and age in continental (**A**) and insular (**B**) populations of *Acanthodactylus schreiberi* with the von Bertalanffy growth model.

Adult specimens in the continental population ranged in age from 3 to six 6 for males (average: 4.65 ± 1.17) and from 3 to 7 years for females (average: 4.36 ± 1.15) (Table 1). Within this group, the average age of males and females did not vary statistically (Mann–Whitney U Test; *p* = 0.38). Cross sections of phalanx 55.6% of the adult specimens showed endosteal resorption, which causes partial erosion of the periosteal bone near the medullary cavity’s margin (Figure 2). The most common age group in the population is the 5th age group, with 12 individuals (28%), followed by the 3rd (26%, *n* = 11) and 4th (23%, *n* = 10) age groups. (Figure 3). Sexual maturity was reached at 2–4 years for males (mean: 2.78 ± 0.13) and females (mean: 2.96 ± 0.11). The expected survival after sexual maturity (ESP) for lizards reaching sexual maturity was calculated to be 6.06 years for males and 5.80 years for females. Males and females had a survival rate (Svr) of 0.82 and 0.81 (Table 2), which means an 82% and 81% survival rate from one year to the later years, respectively. Growth rates were comparable between males (average: 6.14 ± 1.92 mm per year) and females (average: 4.61 ± 1.76 mm per year) within this population (U-test, U = 12, *p* = 0.662). The highest SVL observed in this group for females was greater than the calculated asymptotic SVL (SVLmax, males: 80.47 mm; females: 71.94 mm, Table 2). The Von Bertalanffy equation-predicted growth parameters provide a fit that captures the correlation between SVL and age (Figure 4).

The SVL of male individuals of the continental population ranged from 52.40 mm to 80.11 mm (mean: 70.03 ± 8.58 mm) and from 50.74 to 74.40 (mean: 64.54 ± 7.12) in females. In the continental population, males were found to have statistically greater body length than females (Mann–Whitney U Test; U: 121; *p* < 0.05). Additionally, the SDI was calculated as 0.09, providing a measure of the degree of sexual dimorphism.

The mean body mass of male individuals of the continental population was 7.84 ± 3.06 g, with a range of 2.35 g to 11.32 g. In females, the mean body mass was 4.36 ± 1.15 g, with a range of 1.46 to 7.50 g. The results of the Mann–Whitney U Test revealed that males exhibited a statistically greater body mass than females (U: 82; *p* < 0.01). There were statistical differences in body mass after differences in SVL were controlled (ANCOVA, F = 14.77, df = 1, *p* < 0.05).

In this population, a positive correlation was observed between SVL-age (Pearson Correlation Coefficient; r = 0.574, *p* < 0.001), SVL-body mass (Pearson Correlation Coefficient; r = 0.810, *p* < 0.001), and age-body mass (Pearson Correlation Coefficient; r = 0.504, *p* < 0.01).

### 3.2. Demographic Parameters and Body Size in the Insular Population (Dipkarpaz, Northern Cyprus)

The insular population consisted of adult specimens aged three to six years in males (mean: 4.22 ± 0.97) and two to six years in females (mean: 3.85 ± 1.42) (Table 1). The average age of females and males was similar (Mann–Whitney U Test; *p* > 0.05). The age of the nine juveniles in the insular population was one year (Figure 3B).

The majority of the individuals in the insular population, which made up 55% of the adults (*n* = 16; Figure 3), were 3 and 4 years old. The average age at sexual maturity of males and females in this population was determined as 2.88 ± 0.11 (range 2–3) and 2.85 ± 0.08 (range 2–3) years, respectively. Endosteal resorption was observed in cross sections of 45.22% of the adult specimens of this population.

The calculated ESP for this lizard population was 5.32 years in males and 4.46 years in females. The survival rate (Svr) for males and females was determined to be 0.79 and 0.75 (Table 2). Growth rates between sexes were statistically different, with males growing at an average of 7.25 ± 1.17 mm per year and females at 2.54 ± 0.26 mm per year (U-test, U = 0.0, *p* < 0.01). The predicted asymptotic SVL (SVLmax) was 98.86 mm for males and 107.20 mm for females (Table 2); these values were higher than the maximum observed SVL for males and females (Table 1). The von Bertalanffy growth model provided a reasonable approximation of the relationship between SVL and age in both populations (Figure 4).

The SVL of male individuals of the insular population ranged from 55.00 mm to 76.00 mm and from 42.00 mm to 76.00 mm in females. There was a meaningful disparity in SVL between males (mean: 65.22 ± 6.55 mm) and females (mean: 57.50 ± 10.09 mm) (Mann–Whitney U Test; U = 47.5; *p* < 0.05), supported by a strong SDI of 0.13.

Males in the insular population had a mean body mass of 6.17 ± 1.59 g, ranging from 3.52 g to 7.82 g. Female body mass ranged from 1.14 to 8.80 g, with a mean of 4.21 ± 2.34 g. The males had a significantly higher body mass than the females, according to the findings of the Mann–Whitney U Test (U = 48; *p* < 0.05). When SVL was controlled, the body mass was found to be similar between males and females (ANCOVA, F= 0.632, df = 1, *p* = 0.434).

In the Dipkarpaz population, a positive correlation was observed between SVL-age (Pearson Correlation Coefficient; r = 0.847, *p* < 0.001), SVL-body mass (Pearson Correlation Coefficient; r = 0.938, *p* < 0.001), and age-body mass (Pearson Correlation Coefficient; r = 0.767, *p* < 0.001).

### 3.3. Comparison of the Continental (BOTAŞ) and Insular Populations (Dipkarpaz)

The mean age of males and females in insular individuals was similar to that of their continental counterparts (Two-Way ANOVA; male: F = 0.876; df= 1, *p* > 0.05; female: F = 1.767; *p* > 0.05). Furthermore, when the data set is limited to the collective of all individuals, irrespective of sex, the mean age of the continental population (4.48 ± 1.15 years) was similar to the mean age of the insular population (3.97 ± 1.29 years) (Two-Way ANOVA; F = 2.258; df= 1, *p* > 0.05). Growth rates were similar between the continental and insular populations (Two-Way ANOVA; F = 0.162; df = 1, *p* = 0.692).

The mean SVL of female individuals of the continental population was higher than that of female individuals of the insular population (Two-Way ANOVA; F = 7.5; df = 1, *p* < 0.01). However, the mean SVL value of male individuals was not significantly different between the two populations (Two-Way ANOVA; F = 2.15, df = 1, *p* = 0.156). On the other hand, irrespective of sex, the continental population’s mean SVL was statistically significantly higher than that of the insular group (Two-Way ANOVA; F = 7.74; df = 1, *p* < 0.01).

When SVL was controlled, the body mass of continental females was found to be higher than their insular counterparts (ANCOVA, F = 12.095, df = 1, *p* < 0.01). However, the mean body mass of males in insular individuals was similar to that of their continental counterparts (ANCOVA, F = 0.273, df = 1, *p* = 0.606). Furthermore, when the data set is limited to the collective of all individuals, irrespective of sex, the mean body mass of the continental population (5.89 ± 2.89 g) is analogous to the mean age of the insular population (4.82 ± 2.30 years) (ANCOVA, F = 3.334, df = 1, *p* = 0.072).

A positive correlation was observed between SVL and age values similar to those in both populations. The von Bertalanffy growth model provided a reasonable approximation of the relationship between SVL and age in both populations (Figure 4).

## 4. Discussion

The present study provides valuable insights into the demographic parameters, body size, survival rates, and growth dynamics of *A. schreiberi* continental and insular populations. Further studies are needed to disentangle the adaptive and ecological processes underlying the observed differences. The age structure of both populations revealed non-significant differences in demographic composition. The age ranges of males were identical between the two populations (3 to 6 years), while females in the continental population had a slightly broader range (3 to 7 years) compared to those in the insular population (2 to 6 years). However, this difference is minimal, and overall, the age distributions between the two populations show no substantial variation. This pattern aligns with the findings of other studies, where insular populations often experience increased demographic turnover due to environmental constraints such as limited resources [4,15].

Sexual maturity was achieved at similar ages in both populations (mean: 2.88 years in the continental population; 2.86 years in the insular population). These results are consistent with prior research on lizards, which suggests that age at sexual maturity is relatively conserved across populations within a species but can be influenced by ecological conditions such as food availability and climate [1,14]. Maximum observed ages (7 years in the continental population, 6 years in the insular population) were slightly lower than those reported in other conspecific lizard studies, where individuals can reach ages 9 to 11 years under optimal conditions [21,48,49]. The observed differences likely reflect the ecological pressures unique to each environment, such as predation risk and resource availability.

The survival rates (Svr) and expected survival after sexual maturity (ESP) were generally higher in the continental population compared to the insular one. Males and females in the continental population had Svr values of 0.82 and 0.81, respectively, compared to 0.79 and 0.75 in the insular population. These differences may be influenced by the ecological characteristics of each habitat. The continental site (Botaş, Türkiye) is characterized by higher humidity, abundant freshwater sources, and probably greater diversity and quantity of insects (pers. comm.), which could contribute to increased survival rates and less constrained growth. In contrast, the insular site (Dipkarpaz, Northern Cyprus) is a semi-arid region with limited freshwater availability and lower prey abundance, potentially leading to lower survival rates and growth constraints. The presence of higher environmental stressors in the insular habitat, such as greater seasonal variation in temperature and resource scarcity, may further impact survival. These findings align with studies indicating that continental environments, characterized by more stable and resource-rich conditions, often support higher survival probabilities [15,27]. Endosteal resorption, observed in both populations, underscores age-related bone remodeling, a common phenomenon in reptiles, which environmental stressors can influence [28,50].

In both continental and insular populations, males exhibited a larger mean SVL compared to females, showing a statistically significant difference. Similarly, the Erzin population displayed male-biased sexual size dimorphism (SSD), with males significantly larger than females (mean SVL: 72.71 mm in males, 64.55 mm in females) [21]. The larger male SVL across populations is likely driven by sexual selection pressures, such as competition for mates [14]. Body size differences between the continental and insular populations are evident, particularly among females. The mean SVL of female individuals in the insular population was significantly smaller than their continental counterparts (*p* < 0.01), while no significant difference was observed in males. The increased SVL in continental females could reflect an adaptive strategy to enhance reproductive output, as larger body size is often correlated with higher fecundity in reptiles [14]. Interestingly, the overall mean SVL (regardless of sex) in the insular population was lower than that of the continental, highlighting the potential impact of resource limitations and environmental constraints on growth. The insular site has a drier climate with reduced precipitation and lower insect biomass (pers. comm.)**,** which may restrict growth potential. This finding is consistent with previous studies suggesting that insular populations often exhibit body size shifts due to reduced predation pressure and competition, as well as resource availability [4,5].

Meiri et al. [9] questioned the “island rule” and proposed that it is not a universal model. The island rule describes a trend where small species tend to grow larger on islands while large species tend to become smaller. However, the study demonstrated that this rule does not hold for all mammals or clades, suggesting instead that body size evolution is more clade-specific and context-dependent. The reverse of this rule is called reversed island syndrome and may apply to some lizards. According to this rule, it is assumed that insular lizards will be more aggressive and sexually dimorphic than their continental relatives [6]. Sexual dimorphism indices (SDI) also differed between populations. The continental population exhibited male-biased sexual size dimorphism (SDI: 0.09), while the insular population displayed a stronger male-biased dimorphism (SDI: 0.13). This difference may result from varied ecological pressures, such as sexual selection or differences in resource allocation strategies between sexes. The larger male SVL in both populations might be driven by competition for mates, while the reduced female SVL in the insular population suggests a possible trade-off favoring reproductive investment over body growth [14,51,52].

Growth rates also displayed notable intersexual and interpopulation differences. Males in the insular population exhibited higher growth rates compared to females (*p* < 0.01), aligning with the hypothesis that sexual dimorphism in growth dynamics may arise from differential selection pressures, such as competition for mates in males and energy allocation to reproduction in females [25,29]. In contrast, in the continental population, growth rates were similar between males and females, suggesting that selection pressures shaping growth dynamics may differ between the two environments. The higher resource availability in the continental habitat may allow both sexes to grow at comparable rates. Interestingly, the growth parameters predicted using the von Bertalanffy equation accurately modeled the relationship between age and SVL in both populations, reinforcing the utility of this approach in studying reptilian growth [29]. The von Bertalanffy growth model was used to estimate growth parameters in both populations, as it is widely applied in studies of ectothermic vertebrates. However, we acknowledge that our sample size is limited, which may influence the accuracy of the model’s predictions. To assess the model’s reliability, we evaluated its fit using the coefficient of determination (R^2^), which showed a reasonable approximation of the observed growth patterns.

In the insular population, strong positive correlations were observed between SVL-age, SVL-body mass, and age-body mass. In contrast, the continental population displayed relatively weaker correlations for SVL-age, SVL-body mass, and age-body mass. These findings align with the stronger age-size correlation observed in Erzin females reported by [21], further emphasizing the influence of habitat-specific ecological pressures on growth dynamics. Such comparative analyses are vital for developing conservation strategies tailored to the unique challenges faced by *A. schreiberi* populations in varying environments.

## 5. Conclusions

The observed differences between continental and insular populations underscore the influence of environmental conditions on life-history traits. The higher survival and growth rates in continental populations align with the literature on the benefits of larger, resource-rich habitats. Conversely, the insular population’s adaptations, such as smaller female body size and slower growth, may reflect selective pressures favoring reproductive success and energy conservation. Future research should focus on integrating genetic analyses to explore the evolutionary divergence of these populations and monitor environmental changes that may impact their survival and growth. This study contributes to understanding the adaptive strategies of *Acanthodactylus schreiberi* under varying ecological conditions, providing a foundation for conservation and further exploration of insular-continental dynamics.

## Figures and Tables

**Table 1:** Body size (SVL), longevity, and mean age in *Acanthodactylus schreiberi* specimens from the continental and insular sites.

Location	SVL (mm)		Age (Years)		Longevity (Years)	
	Male	Female	Male	Female	Male	Female
Continental Population, Türkiye	70.03	64.54	4.65	4.36	6	7
Insular Population, Cyprus	65.22	57.50	4.22	3.85	6	6

**Table 2:** Descriptive statistics of growth rate (mm per year), growth coefficient (k), ESP, and Sr in the studied populations of *Acanthodactylus schreiberi* adults from Türkiye and Cyprus. N—number of specimens, SE—standard error of the mean, ESP-adult life expectancy, Svr—survival rate, SDI—sexual dimorphism index.

Group	Sex	N	Growth Rate ± SE	k	SVLmax	ESP	Svr	SDI
Türkiye	Males	17	6.14 ± 1.92	0.41	80.47	6.06	0.82	0.09
	Females	25	4.61 ± 1.76	0.53	71.94	5.80	0.81	
Cyprus	Males	9	7.25 ± 1.17	0.19	98.86	5.32	0.79	0.13
	Females	20	2.54 ± 0.26	0.05	107.2	4.46	0.75	

## Data Availability

All data generated or analyzed during this study are included in this manuscript.

## Supplementary Materials

The following supporting information can be downloaded at https://www.mdpi.com/article/10.3390/ani15040474/s1, Table S1: Museum Collection Data of *Acanthodactylus schreiberi*.
