# Supplementary material for: Continental vs. Insular: Demographic and Growth Patterns in Acanthodactylus schreiberi
Source: Animals (Basel). 2025 Feb 7;15(4):474. doi: 10.3390/ani15040474 (PMC11852165; doi:10.3390/ani15040474)
Supplement: Supplementary file 1 [file animals-15-00474-s001.zip › animals-3431844-supplementary.pdf]

Supplementary File

Continental vs. Insular: Demographic and Growth Patterns in *Acanthodactylus schreiberi*

Büşra Kara [1], Mehmet Zülfü Yıldız [2,*], Deniz Yalçınkaya [3] and Abdullah Altunışık [4,*]

[1] Biology Department, The Institute of Graduate Studies, University of Recep Tayyip Erdoğan, Rize 53100, Türkiye; busra_kara24@erdogan.edu.tr
[2] Zoology Section, Biology Department, Faculty of Arts and Sciences, Adıyaman University, Adıyaman 02040, Türkiye
[3] Medical Laboratory Techniques Program, Department of Medical Services and Imaging, Vocational School, Toros University, Mersin 33140, Türkiye; dyalcinkaya@yahoo.com
[4] Biology Department, Faculty of Arts and Sciences, University of Recep Tayyip Erdoğan, Rize 53100, Türkiye
* Correspondence: zulfuyildiz21@gmail.com (M.Z.Y.); abdullahaltunisik@gmail.com or abdullah.altunisik@erdogan.edu.tr (A.A.)

Table S1. Museum Collection Data of *Acanthodactylus schreiberi*

**Continental Samples, Türkiye**
ZDEU 006/1999: (n=16), 8 (♂♂), 8 (♀♀), Burnaz/Erzin/Hatay, 11.07.1999, Leg. M. TOSUNOĞLU, K. AKMAN
ZMADYU 008/2005: (n=4), 4 (♂♂), Burnaz/Erzin/Hatay, 01.09.2005, Leg. D. YALÇINKAYA.
ZMADYU 009/2005: (n=7), 1 (♂♂), 6 (♀♀), Burnaz/Erzin/Hatay, 03.08.2005, Leg. D. YALÇINKAYA.
ZMADYU 133/2007: (n=2), 1 (♂), 1 (juvenile), Burnaz/Erzin/Hatay, 30.04.2007, Leg. B. GÖÇMEN, M. Z. YILDIZ, B. AKMAN, D. YALÇINKAYA.
ZMADYU 166/2007: (n=14), 3 (♂♂), 11 (♀♀), Burnaz/Erzin/Hatay, 27.05.2007, Leg. B. GÖÇMEN, M. Z. YILDIZ, B. AKMAN, D. YALÇINKAYA.
**İnsular Samples, Cyprus**
ZMADYU 012/2003: (n=3), 2 (♂♂), 1 (♀), Cyprus, 01.07.2003, Leg. B. GÖÇMEN
ZMADYU 033/2007: (n=33), 7 (♂♂), 18 (♀♀), 8 (juvenile) Altınkum/Karpas/Cyprus, 04.04.2007, Leg. B. GÖÇMEN, M. Z. YILDIZ, B. AKMAN, D. YALÇINKAYA, N. KAŞOT.
ZMADYU 222/2007: (n=1), 1 (♀), Derinya/Larnaca/Cyprus, 19.09.2007, leg B. GÖÇMEN, N. KAŞOT.
ZMADYU 224/2007: (n=1), 1 (juvenile), Nicosia-S. Cyprus [9], 20.09.2007, leg B. GÖÇMEN, N. KAŞOT.
